# Treatment of Arsenite Intoxication-Induced Peripheral Vasculopathy with Mesenchymal Stem Cells

**DOI:** 10.3390/ijms19041026

**Published:** 2018-03-29

**Authors:** Yi-Hung Chiang, Chai-Chin Lin, Yen-Chung Chen, Oscar K. Lee

**Affiliations:** 1 Institute of Clinical Medicine, National Yang-Ming University, Taipei 11221, Taiwan; 2 Department of Orthopaedics, National Yang-Ming University Hospital, Yilan 260, Taiwan; 3 Department of Biotechnology and Animal Science, National Yilan University, Yilan 260, Taiwan; Lincc@niu.edu.tw; 4 Department of Pathology, National Yang-Ming University Hospital, Yilan 260, Taiwan; 999liquor999@gmail.com; 5 Stem Cell Research Center, National Yang-Ming University, Taipei 11221, Taiwan; 6 Taipei City Hospital, Taipei 10341, Taiwan; 7 Department of Medical Research, Taipei Veterans General Hospital, Taipei 11217, Taiwan; 8 Department of Orthopaedics and Traumatology, Taipei Veterans General Hospital, Taipei 11217, Taiwan

**Keywords:** arsenite, peripheral vascular disease, mesenchymal stem cells

## Abstract

Arsenite (As), a notorious toxic metal, is ubiquitously distributed in the earth and poses a serious threat to human health. Histopathological lesions of As intoxication are known as thromboangiitis obliterans, which are resistant to current treatment and often lead to lower limb amputation. In this study, we attempt to find that treatment with mesenchymal stem cells (MSCs) may be effective for As-induced vasculopathy. We first conducted an in vitro study with a co-culture system containing human MSCs and human umbilical vein endothelial cells (HUVECs) and treated individual and co-cultured cells with various concentrations of arsenite. We also designed an in vivo study in which Sprague Dawley (SD) rats received periodic intraperitoneal (IP) injections of 16 ppm arsenite for 12 weeks. MSCs were harvested from BALB/c mice that were transplanted via tail vein injection. We found that there was significantly higher cellular viability in human mesenchymal stem cells (hMSCs) than in HUVECs under concentrations of arsenite between 15 and 25 μM. The Annexin V apoptosis assay further confirmed this finding. Cytokine array assay for As-conditioned media revealed an elevated vascular endothelial growth factor (VEGF) level secreted by MSCs, which is crucial for HUVEC survival and was evaluated by an siRNA VEGF knockdown test. In the in vivo study, we demonstrated early apoptotic changes in the anterior tibial vessels of As-injected SD rats with a Terminal deoxynucleotidyl transferase dUTP nick end labeling (TUNEL) assay, but these apoptotic changes were less frequently observed upon MSCs transplantation, indicating that the cytoprotective effect of MSCs successfully protected against As-induced peripheral vasculopathy. The feasibility of MSCs to treat and /or prevent the progression of As-induced vasculopathy is justified. Further clinical studies are required to demonstrate the therapeutic efficacy of MSCs in patients suffering from As intoxication with vasculopathy.

## 1. Introduction

Toxic metals pose serious adverse effects to the environment and human health. Among these metals, arsenite (As) is most commonly found due to its ubiquitous distribution in the earth’s crust [1]. For decades, most endemic arsenic exposures in humans have come from environmental sources and threatened millions of people in various regions worldwide, including Argentina, Bangladesh, Chile, China, Hungary, the United States and Taiwan [1]. Recently, the adverse health effects resulting from chronic arsenite exposure are thought to be transmitted through drinking water, worldwide food distribution, smoking and global cosmetics [2]. Arsenite pollution poses a global threat and is never-ending.

Chronic exposure to arsenite leads to skin lesions, neurological dysfunction, atherosclerosis and cancer [3]. Those catastrophic insults threaten health. Arsenite often targets the vascular system as an initiation of its toxicity, and the damage to the vascular system plays an important role in mediating pathological changes in specific target organs [4]. It has been reported that the generation of reactive oxygen species (ROS) during arsenic metabolism orchestrates arsenite-induced injury [5]. Persistent ROS elevation has a deleterious effect on endothelial cells and cells may lose antioxidant defenses, leading to apoptosis [6,7]. The causative effect of arsenite on the peripheral vascular system gives rise to histologic lesions known as thromboangitis obliterans and/or arteriosclerosis obliterans. Clinically, patients are noted with artery insufficiency, chronic ulceration and gangrene. A majority of lesions are located on lower extremities and thus the condition is called black foot disease, a prevalent endemic disease in Taiwan [8]. Treatment for such troublesome complications has been researched for decades and toxic metal-induced vascular diseases are still difficult to treat, often eventually leading to amputation.

There are few options for the treatment of arsenite-induced vascular injury. Peripheral arterial disease exhibits a similar aggressive clinical presentation and various therapeutic strategies have been established to improve circulation. Potential cell therapies are based on the stimulation of angiogenesis with extracellular or cellular components, including endothelial progenitor cells and stem cells [9,10,11]. The majority of these treatments have used either bone marrow-derived or peripheral mononuclear cells. Those progenitors can restore tissue vascularization after ischemic events in limbs [12]. However, in order to expand stem cell treatment to arsenite intoxication, it is critical to understand whether these cells tolerate the toxicity of arsenite.

Mesenchymal stem cells (MSCs), distinct from hematopoietic stem cells, have long been identified in the bone marrow stoma as colony-forming unit-fibroblasts (CFU-F) [13] and have the capability of differentiation into tissues of mesodermal origin [14,15,16,17]. They exhibit characteristics of stem cells and have been expected to bring substantial benefit for the treatment of a variety of diseases and injuries. Potential clinical applications of human MSCs (hMSCs) have been reported in recent years [18,19,20,21,22]. It is well documented that mechanisms mostly derived from MSCs can orchestrate tissue repair through the secretion of a broad range of bioactive factors and those trophic factors can modulate cellular activity [23,24]. At the same time, as perivascular niche cells, MSCs have a great capability to modulate endothelial function and enhance angiogenesis [25,26].

MSCs may be an ideal candidate for cellular therapy for arsenite-induced vascular disease due to their potential for promoting angiogenesis. It has been reported that low concentrations of As (0.1–5 μM) can inhibit CD34^+^ hematopoietic stem cell proliferation and differentiation into various hematological cell lineages [27]. On the other hand, MSCs have been found to tolerate higher concentrations of arsenite and are more resistant to arsenite exposure [28]. For this reason, we chose MSCs as therapeutic cells in the scenario of arsenite intoxication. We conducted in vitro and in vivo experiments to verify the therapeutic efficacy of MSCs with a co-culture system and a rat animal model. Our hypothesis is that MSCs can recover the damage of endothelial cells caused by As.

## 2. Experimental Results

**Figure 1:**
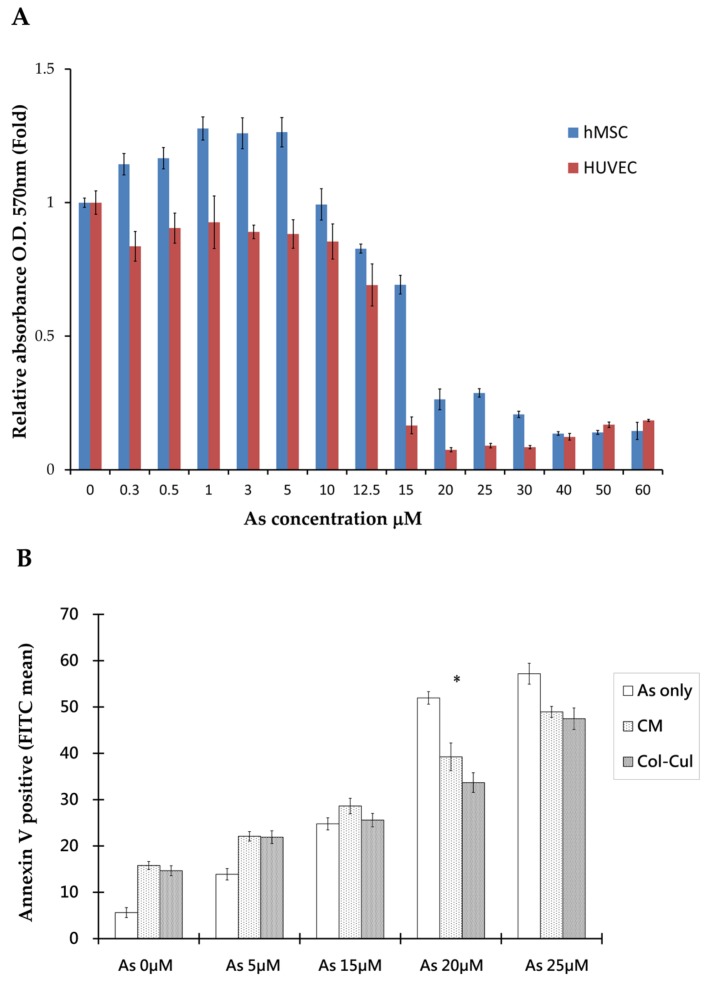
(**A**) MTT assays for human umbilical vein endothelial cells (HUVECs) and human mesenchymal stem cells (hMSCs) under arsenite treatment show a distinct difference in the survival rate between HUVECs and hMSCs. There are significantly higher survival rates in hMSCs than in HUVECs under high concentrations of arsenite between 15 to 25 μM; (**B**) An apoptosis flow cytometry study reveals significant differences in apoptosis rates between HUVECs treated with arsenite, HUVECs in conditioned medium and HUVECs and hMSCs co-cultured under an arsenite concentration of 20 μM. (* *p* < 0.05), MTT assay—(3-[4,5-dimethylthiazol-2-yl]-2,5-diphenyltetrazolium bromides). Col-cul—co-cultured HUVEC and hMSCs. CM—conditioned medium.

The (3-[4,5-dimethylthiazol-2-yl]-2,5-diphenyltetrazolium bromides) MTT assay was used to assess cellular viability after arsenite treatment. The results showed that there were distinct viability differences between hMSCs and human umbilical vein endothelial cells (HUVECs) when treated with arsenite for 48 h. By stepping up the dosage of arsenite, we found that there were significantly higher survival rates in hMSCs than in HUVECs at a high concentration of arsenite between 15 to 25 μm. In addition, the calculated lethal concentration 50% (LC50) was 18.52 μM for hMSCs and 12.79 μM for HUVECs, as shown in Figure 1A. hMSCs can resist more arsenite toxicity than HUVECs. From the morphology of cells under the microscope, hMSCs distributed homogenously and exhibited spindle cell morphology at concentrations as high as 10 μM arsenite.

**Figure 2:**
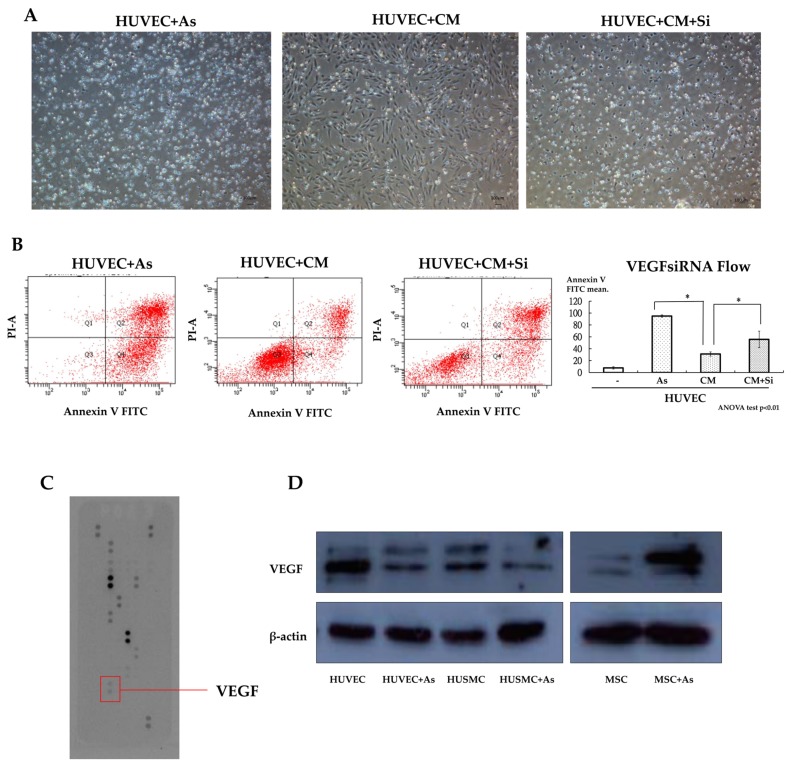
(**A**) Microscopic view of a culture dish with 20 µM arsenite treatment for 48 h shows obvious differences in the morphology and viability of HUVECs between cells cultured alone and those co-cultured with hMSCs. The left picture shows that when we delivered VEGF (vascular endothelia growth factor) siRNA into hMSCs, the harvested siRNA conditioned medium could not rescue the HUVECs and more cell death was found compared with the middle picture. (Scale bar 100 µm) (**B**) Apoptosis flow cytometry results show fewer HUVECs deviating to zone 1 and zone 4 (Annexin V positive) when cultured with conditioned media from hMSCs. This phenomenon is abolished when we deliver siRNA into hMSCs. (* *p* < 0.05) (**C**) Cytokine array assay reveals an elevated VEGF level in conditioned media with hMSCs treated with As compared with that in normal medium. (**D**) Western blot experiments show the same result. More VEGF was expressed upon As treatment in the conditioned medium than in the normal medium (hMSCs only). CM—conditioned medium with HUVECs treated with 20 µm As. CM+Si—conditioned medium with HUVECs and the addition of VEGF SiRNA. hMSCs—human mesenchymal stem cells. HUSMCs—human umbilical vein smooth muscle cells.

From the co-culture of hMSCs and HUVECs with transwell plates, differences in the morphology and viability of HUVECs between cultures alone or co-cultured with hMSCs at 20 µM arsenite are shown in Figure 1B and Figure 2A. Under such circumstances, more HUVECs survived when co-cultured with hMSCs. Similar effects could also be seen when HUVECs were grown with arsenite-treated conditioned media. Apoptosis of HUVECs was detected under treated and untreated conditions using Annexin V apoptosis assay and flow cytometric studies (Figure 1B). Data showed that in the untreated group, 51.7% HUVECs underwent apoptosis, as shown with positive Annexin V staining. In contrast, when HUVECs were treated with conditioned medium from hMSCs for 48 h, only 33.6% of HUVECs exhibited positive staining for Annexin. This difference was significant (*p* < 0.05), as shown in Figure 1B. From this study, we could characterize the effect of hMSCs, which are capable of preventing apoptosis in HUVECs caused by As intoxication.

By using the cytokine array assay, it was found that the conditioned medium with hMSCs treated with As exhibited a significant increase in vascular endothelial growth factor (VEGF) levels compared with normal medium, as shown in Figure 2C. Western blot analyses showed the same result when we compared the medium subjected to As treatment with the untreated medium, as shown in Figure 2D. We found that As treatment could trigger the secretion of VEGF from hMSCs. To demonstrate the effect of VEGF, hMSCs were transfected with VEGF siRNA to abrogate VEGF secretion and the knockdown effect was confirmed by an (enzyme-linked immunosorbent assay) ELISA. We analyzed the function of VEGF knockdown hMSCs with the Annexin V apoptosis assay and repeated the As treatment experiment as mentioned before. The results showed that when we delivered VEGF siRNA into hMSCs, there was a significant increase in the apoptosis of HUVECs treated with harvested conditioned medium from knocked-down hMSCs, as shown in Figure 2A,B. From the results of the Annexin V apoptosis assay, we found fewer HUVECs deviating to zone 1 and zone 4 (Annexin V positive) when cultured with conditioned medium from hMSCs. This phenomenon was abolished when we delivered siRNA into hMSCs (Figure 2B). Transfected hMSCs lose their protective and therapeutic effects due to decreases in the secretion of VEGF.

**Figure 3:**
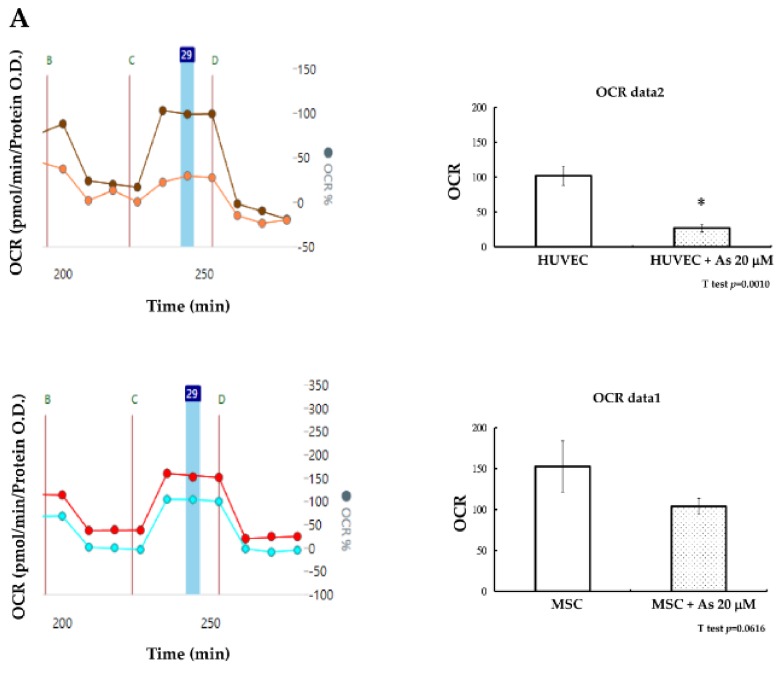

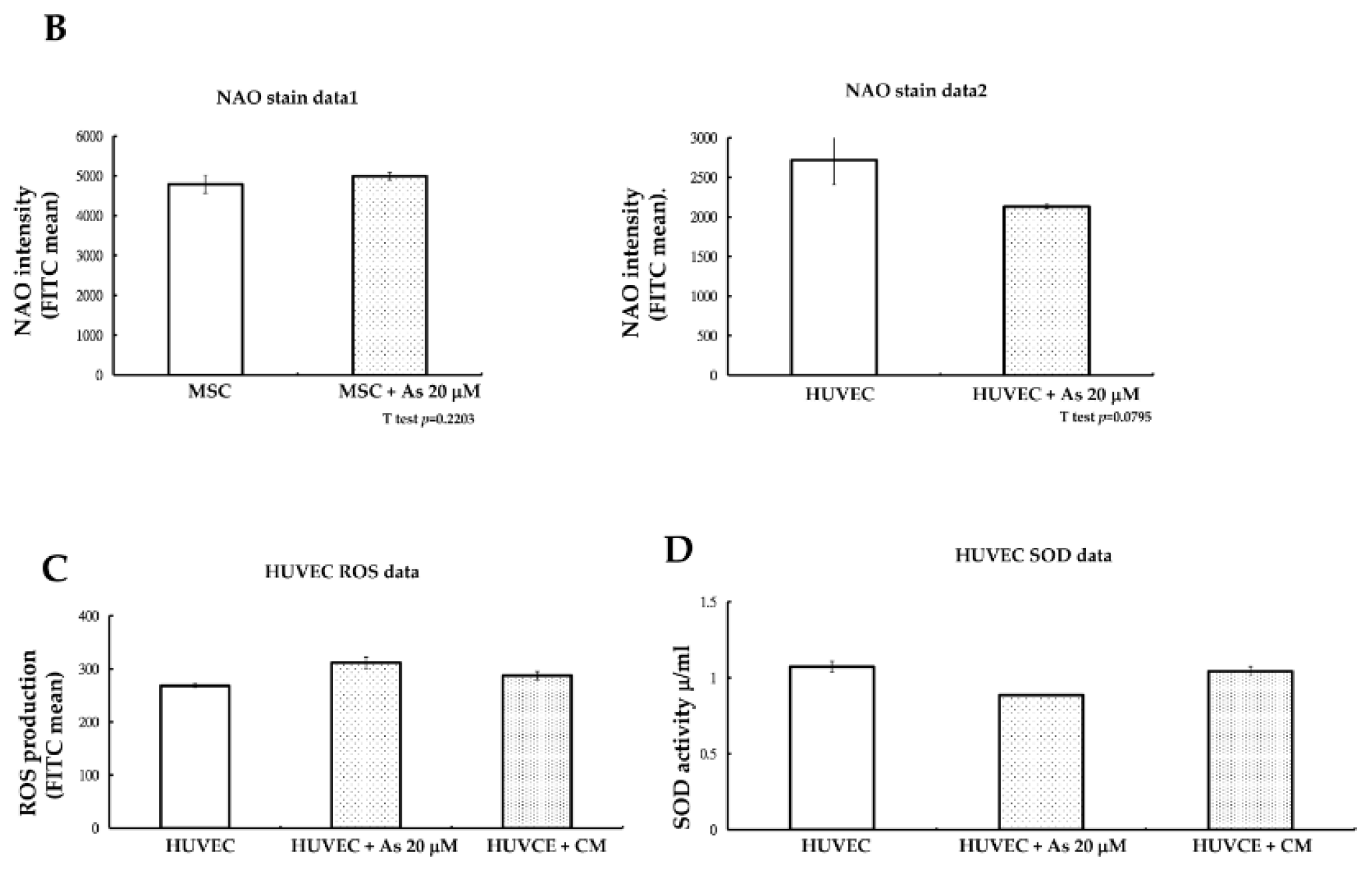
(**A**) A mitochondrial maximum oxygen consumption rate (OCR) test shows that with Seahorse XF-24, the OCR of hMSCs remains constant without significant changes before or after treatment with As. However, the OCR of HUVECs decreases significantly at a high concentration of As and the difference is statically significant (* *p* < 0.05). Cells were sequentially treated with oligomycin (line B), carbonyl cyanide p-trifluoromethoxy-phenylhydrazone (FCCP) (line C), as well as rotenone and antimycin A (line D). Maximal respiratory rate was measured after cells were treated with FCCP (blue bar zone 29). Dark orange line—HUVEC, Light orange line—HUVEC with As 20 μM. Red line—MSC, Blue line—MSC with As 20 μM. (**B**) A mitochondrial mass nonyl acridine orange (NAO) fluorescence assay demonstrates that under 20 µM As, the NAO green fluorescent protein (GFP) mean in HUVECs decreased compared with that in normal HUVECs. hMSCs maintain a constant mean before and after treatment with As. (**C**) A reactive oxygen species (ROS) assay demonstrates that ROS levels declined slightly in HUVECs treated with conditioned medium compared with those in cells treated with As for only 48 h. (**D**) An antioxidant superoxidase dismutase (SOD) assay shows increased SOD activity in HUVECs treated with conditioned medium compared with that in cells treated with As only. hMSCs—human mesenchymal stem cells. HUVECs—human umbilical vein endothelial cells.

How hMSCs are able to sustain the insults of As was further elucidated based on the investigation of cellular energy metabolism. Mitochondria dysfunction can contribute to cell apoptosis and cellular oxygen consumption has been recognized as a fundamental measure of mitochondrial function [29]. Seahorse XF-24 (Seahorse Bioscience Inc., Billerica, MA, USA) was used to measure the maximum oxygen consumption rate (OCR) of hMSCs and HUVECs after treatment with As. The oxygen consumption rate of hMSCs remained constant without significant changes before or after treatment with As. We observed that the decline of functional mitochondria in hMSCs under high concentrations of As (20 μM was not significant (*p* = 0.061). In contrast, the oxygen consumption rate of HUVECs decreased significantly under high concentrations of As (Figure 3A). Mitochondrial mass was checked at the same time and we found that there were also no variances in nonyl acridine orange (NAO) fluorescent density between hMSCs alone and hMSCs treated with 20 μM As. On the other hand, NAO fluorescence density declined in HUVECs when comparing cells with or without As treatment. We concluded that hMSCs could preserve more functional, active mitochondria and withstand a higher dose of arsenite.

Reactive oxygen species (ROS) generated under high As concentrations often lead to the apoptosis of endothelial cells. We further investigated whether the toxic ROS could be reduced after HUVECs were treated with hMSCs conditioned media. After treatment with hMSCs conditioned media for 48 h, an ROS assay with flow cytometry demonstrated that ROS levels (fluorescein isothiocyanate FITC mean) in HUVECs declined slightly compared with those in cells treated with As only (Figure 3A). The antioxidant capacity of HUVECs under conditioned medium treatment was then checked. We measured superoxidase dismutase (SOD) activity and the results showed that SOD activity increased under conditioned medium treatment, as shown in Figure 3B. However, the variability was not statistically significant. 

**Figure 4:**
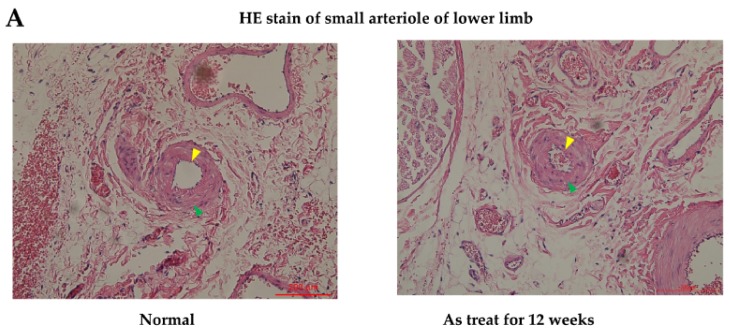

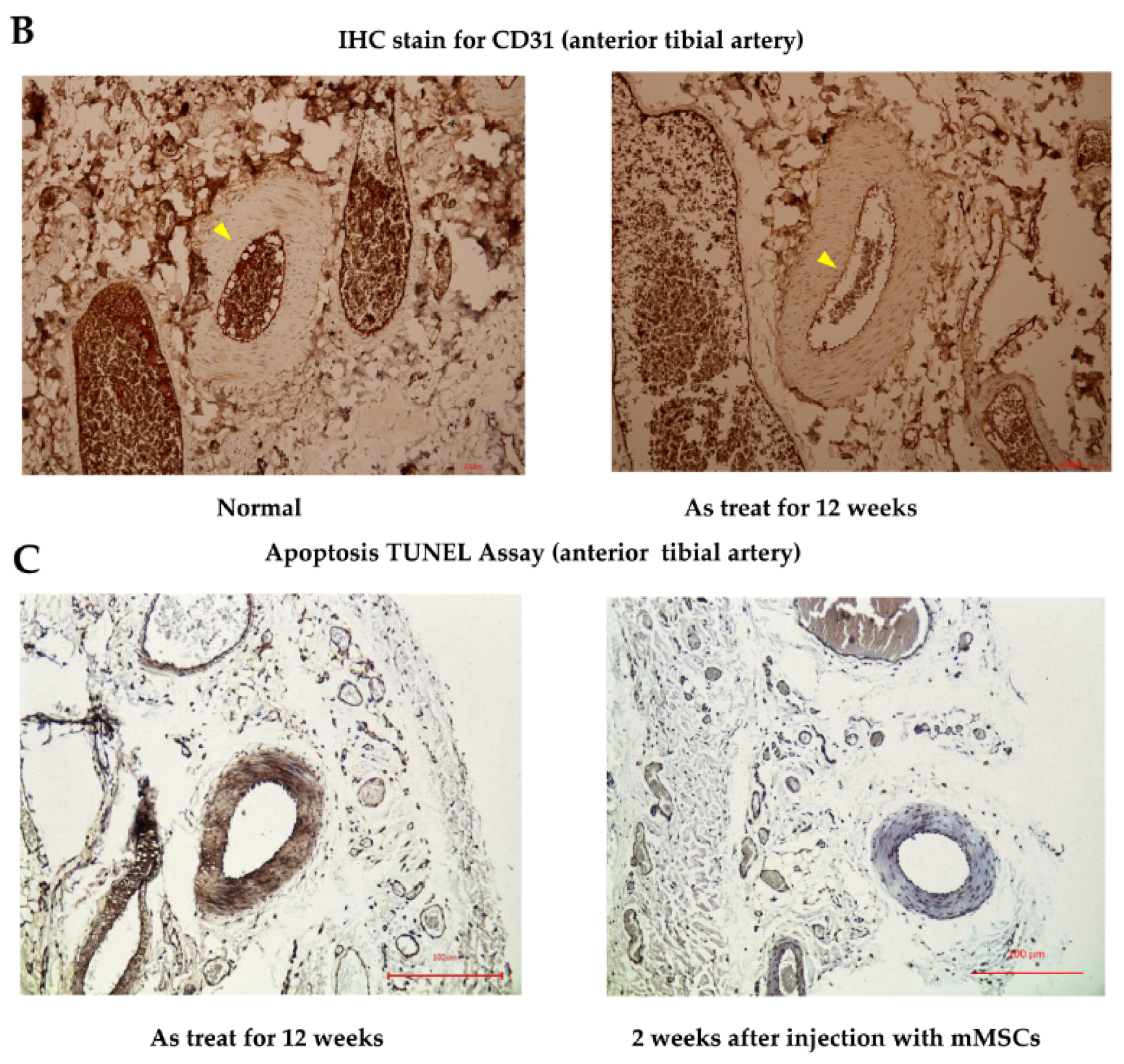
(**A**) HE (hematoxylin and eosin) staining for a small arteriole from the anterior lower limb of an SD rat shows a decrease in the lumen diameter of the arteriole (yellow arrow) and an increase in the number of inflammatory cells infiltrating the smooth muscles of vessel walls (green arrow) when compared with a wild-type untreated rat (left). Scale bar 200 μm. (**B**) IHC CD31 staining for vessels reveals a decrease in staining intensity in the As-treated group compared with that in the wild-type SD rat group (yellow arrow). Scale bar 200 μm. (**C**) A TUNEL assay shows numerous TUNEL-positive tissues induced by As in the control group. Compared with the control specimens, mMSCs-injected SD rat specimens show a decrease in TUNEL reactions, especially in regions containing anterior tibial vessels. Scale bar 100 μm. SD rat—Sprague Dawley rat. IHC—immunohistochemistry. CD31 stain—platelet endothelial cell adhesion molecular stain. TUNEL assay—terminal deoxynucleotidyl transferase dUTP nick end labeling assay. mMSCs—mouse mesenchymal stem cells.

In the in vivo study, hematoxylin and eosin stain (HE staining) for vessels harvested from the anterior compartment of the lower limbs of Sprague Dawley (SD) rats showed that there were no obvious histological differences in the anterior tibial artery and vein from the mouse mesenchymal stem cells (mMSCs)-injected and control groups (after 12 weeks of treatment). However, in the arterioles of the control group, as shown in Figure 4A, mild pathological changes were observed, with a decrease in the diameter of the arteriole lumen and an increase in inflammatory cells infiltrating the smooth muscles of vessel walls when compared with normal specimens. The diameter of the arteriole decreased from 100 to 65 μm between normal and As-treated rats. Platelet endothelial cell adhesion molecular (CD31) staining for vessels revealed a decrease in staining intensity in control rats compared with that in wild-type SD rats. Apoptotic ratios of tissues were analyzed by a terminal deoxynucleotidyl transferase dUTP nick end labeling assay (TUNEL assay), which showed that there were significantly more TUNEL-positive tissues induced by As in the control groups. Compared with the control rat specimens, mMSCs-injected SD rat specimens showed decreases in TUNEL staining, especially in regions containing anterior tibial vessels. This finding indicates that mMSCs injection could reverse the early apoptotic changes in vessels induced by As intoxication.

**Figure 5:**
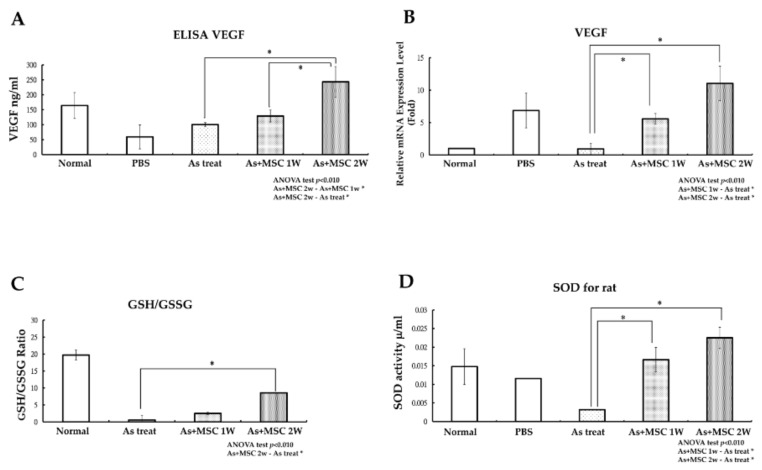
(**A**) An in vivo ELISA VEGF test demonstrates that VEGF levels significantly increase in blood samples drawn from SD rats with serial mMSCs (mouse MSCs) injections for two weeks (* *p* < 0.01). (**B**) An in vivo VEGF qPCR test shows increasing gene expression in rats after serial mMSCs injections for two weeks. (* *p* < 0.05). (**C**) An in vivo GSH/GSSG assay shows that the GSH/GSSG ratio increases in experimental groups subjected to serial mMSCs injections for two weeks compared with that in the As-treated group (* *p* < 0.05). (**D**) In vivo antioxidant superoxidase dismutase (SOD) assay shows increased SOD activity in experimental groups subjected to serial mMSCs injections for two weeks compared with that in the As-treated group (* *p* < 0.05). VEGF—vascular endothelial growth factor. mMSCs—mouse mesenchymal stem cells. PBS—phosphate buffered saline. MSC 1w—mouse MSCs injected for one week. MSC 2w—mouse MSCs injected for two weeks. GSH—reduced glutathione. GSSG—oxidized glutathione.

To investigate VEGF expression in vivo, serum levels of VEGF were evaluated with an ELISA test and VEGF gene expression levels in the lower limb blood vessels of rats were checked with a qPCR test. Compared to the control group, SD rats injected with arsenite without mMSCs treatment and experimental rats that received serial mMSCs injection for two weeks demonstrated a significant elevation in blood VEGF levels, as shown in Figure 5A. VEGF levels were elevated significantly in blood samples drawn from SD rats with serial mMSCs injection for two weeks. Similar findings in the VEGF gene expression levels were achieved with the qPCR test (Figure 5B). We also checked oxidative stress in vivo with the measurement of reduced/oxidized glutathione (GSH/GSSG) levels and the detection of antioxidant SOD levels. Compared with the experimental rats serially treated with mMSCs for two weeks, SD rats exposed to arsenite without mMSCs treatment exhibited a decline in GSH/GSSG ratio, as shown in Figure 5C. The increase in the GSH/GSSG ratio indicated that injection of mMSCs could decrease oxidative stress in the tissues of experimental groups. During the same time period, levels of SOD were also elevated in the experimental groups, as shown in Figure 5D. Treatment of MSCs could prevent further increase of oxidative stress in arsenite intoxication and could reverse the apoptosis of cells lining the lumen of lower limb vessels.

## 3. Discussion

The negative effects of toxic arsenite on human health have been widely studied. However, there are no effective therapies to reverse its pathology. The paucity of appropriate treatments is part of the grim struggle in the conflict with arsenite intoxication. In this study, we demonstrated that MSCs can recover the early pathological change of small vessels from arsenite intoxication. Vascular tissues are an empirical target of arsenite for both the initiation of injury and disease progression. The disruption of the vascular system can lead to a variety of pathologies of tissues in arsenite intoxication and, therefore, we focused on the function of endothelial cells. From our studies, MSCs are able to prevent the apoptosis of endothelial cells and can preserve the integrity of small vessels. We chose MSCs as therapeutic cells because, compared with endothelial cells, they can endure arsenite toxicity and survive under higher concentrations of arsenite. There have only been a few reports of different responses to arsenite toxicity between progenitor cells and differentiated adult cells. Based on our MTT assay, the LD50 for hMSCs under arsenite treatment was 14.52 μM, which is much higher than the LD50 of HUVECs. According to the research of precise As_2_O_3_ (iAs) toxicity to CD34^+^ cells, the author found that iAs with a concentration between 0.1 and 5 can inhibit the proliferation of CD34^+^ cells and more than 70% of CD34^+^ cells were detected as necrotic cells when the iAs concentration was more than 5 M [27]. So, CD34^+^ cells cannot be the candidates for cells therapy in As-induced vasculopathy. In another study on As-induced apoptosis of hMSCs, the author found that with less than 5 M iAs, there was no obvious change in MSCs viability [28]. The distinct viability differences between adult cells and MSCs during As intoxication are consistent with our results.

We tried to verify the mechanism of how hMSCs can survive in high concentrations of As. Mitochondrial function is inextricably linked with cell apoptosis and cellular oxygen consumption is increasingly recognized as a fundamental measurement of mitochondrial function [29,30]. In our study, mitochondrial bioenergetics in arsenite exposure, especially at a high concentration of As (20 μM) in hMSCs, was evaluated with the Seahorse extracellular flux analyzer SFX-20. Seahorse XF-20 can provide real-time measurements of cellular oxygen consumption and provide assessments of the cellular bioenergetics function of cells [31]. Our results show that hMSCs can preserve less dysfunctional mitochondria even in the harsh environment provoked by high As concentration (20 μM). Mitochondrial mass can be a surrogate marker for mitochondrial biogenesis [32,33], as was measured with NAO staining. The results of the fluorescent assay show similar results to cellular oxygen consumption rates. According to previous research, prolonged exposure of vascular tissues (vascular smooth muscle cells) to inorganic arsenite (iAsIII) can induce a reduction in mitochondrial content, cause the fragmentation of mitochondria and lead to a decline in mitochondrial respiration [34]. From our experimental results, we found that hMSCSs exhibit less of a decline in mitochondrial mass during As intoxication, which may be why hMSCs presented fewer differences in oxygen consumption rate (OCR) in high As concentration in SXF-20 experiment. From our experimental results, we found that hMSCs can preserve more functional mitochondria in a high concentration of As and thus escape the toxic effects of As.

MSCs can secrete various cytokines when confronted with high concentrations of As. MSCs serve as a reservoir for various cytokines and can secrete cytokines in response to tissue damage [35,36]. Complex cross talk occurred between injured tissues and MSCs and then MSCs were activated to express various levels of therapeutic protein. The mechanisms underlining MSCs cell therapy demonstrate that the paracrine effects of MSCs are dominant [37,38,39]. Due to the complexity of the paracrine effect of MSCs, we choose to investigate the conditioned medium obtained from As-treated MSCs. From our cytokine array data, when comparing various cytokines exerted by hMSCs with or without As treatment, VEGF was found to play an important role and was most significantly affected after As treatment. VEGF has long been recognized as an important factor for endothelial cell proliferation and migration, which can promote angiogenesis [40,41] and inhibit the apoptosis of endothelial cells [42]. It also has been reported that VEGF can decrease the level of cellular apoptosis through activating phosphokinase B (Akt) both in renal tubular cells [39] and aortic endothelium cells [43]. Introducing anti-VEGF antibodies can adversely increase cellular apoptosis [39]. We provided further evidence of the anti-apoptotic effect of VEGF by knockdown experiments. HUVECs cannot maintain their growth and survival in the same hMSC conditioned medium at the same concentration of As (20 μM), in which VEGF was knocked down. These results are consistent with a previous report on cisplatin-induced renotoxicity in mice [39]. The paracrine effects of MSCs that can release various cytokines in response to tissue damage are well known; however, we are the first to demonstrate that MSCs can secrete abundant VEGF under As intoxication.

In this study, we generated an animal model of As intoxication through weekly IP injection with As for SD rats for 12 weeks. The final blood As concentrations calculated are 108.61 ± 16.25 μg/L which is very similar to the As blood concentration reported in high exposure areas [44]. We harvest anterior tibial vessels to find early pathological and apoptotic changes with HE staining and TUNEL assay. The early pathological changes in small arteriole are consistent with histological pictures of thromboangitis obliterans. After two weeks of treatment with MSCs harvested from BALB/c mice, we revealed a recovery of vessels from apoptotic changes as proved by TUNEL assays. We also demonstrated a significant elevation in blood VEGF levels after two weeks of mMSCs injection by ELISA. This finding indicated that allogeneic transplantation with mMSCs in SD rats could reverse the early apoptotic changes of peripheral vessels. It is well known that the generation of reactive oxygen species (ROS) under As exposure contributes to cellular apoptosis [6]. Oxidative stress leads to mitochondria dysfunction and in turn perpetuates cellular damage, which was found in our in vitro study. It has been reported that the anti-apoptotic effects of growth factors are based on their abilities to prevent oxidative stress by increasing the activity of antioxidant and normalizing mitochondria functions [45]. In the context of our present in vivo study, GSH/GSSG levels decreased after two weeks of treatment with mouse MSCs and the antioxidant enzyme SOD levels were elevated in the same time. Such effects may come from the contribution of secreted growth factors by mMSCs according to the report of previous research [45]. A similar phenomenon was also found in the in vitro studies, but the differences were not so significant. Therefore, we believe that repeated treatment and a longer time frame may be required to support the further reinforcement of antioxidant enzyme levels and a solid decrease in ROS levels. In this study, human MSCs have been tested and their potential therapeutic efficacy in As-induced vasculopathy has been cleared demonstrated. While this proof-of-concept pre-clinical study showed promising results, other factors, such as the cost and applicability of allogeneic use of hMSCs, must be taken into consideration. There are still great challenges of manufacturing a ready supply of highly defined transplantable hMSCs. A stringent compliance of regulations is essential for successful clinical translation.

## 4. Materials and Methods

### 4.1. Maintenance and Expansion of Msenchymal Stem Cells (MSCs) and Human Umbilical Vein Endothelial Cells (HUVECs)

Commercially available human MSCs (hMSCs) were used in this study (Steminent Therapeutics Inc., Taipei, Taiwan). Their abilities to differentiate into osteoblasts, chondrocytes and adipocytes were confirmed. The isolation of murine MSCs from the bone marrow of BALB/c mice was performed with our previously reported method [32]. Commercially available HUVECs, which were isolated from human umbilical veins, were purchased from ScienCell (Carlsbad, CA, USA). As adherent cells reached approximately 50% to 70% confluence, they were detached with 0.25% trypsin-EDTA (ethylenediaminetetraacetic acid; Gibco, Grand Island, NY, USA), washed twice with phosphate-buffered saline (PBS; Sigma-Aldrich, St. Louis, MO, USA), then centrifuged at 1000 rpm (200× *g*) for 5 min and reseeded at a ratio of 1:3 under the same culture conditions.

### 4.2. Culture Medium

hMSC expansion medium consisted of mesenchymal stem cell growth medium (MesenPRO; Gibco), 100 U penicillin, 1000 U streptomycin and 2 mM l-glutamine (Gibco). Murine MSCs were cultured in low-glucose Dulbecco’s Modified Eagle’s medium (LGDMEM; Sigma-Aldrich, St. Louis, MO, USA) supplemented with 10% fetal bovine serum (FBS; Thermo Fisher Scientific, Waltham, MA, USA) and 1% penicillin-streptomycin-glutamine (PSG; Thermo Fisher Scientific). We used endothelial cell medium (ECM; Sigma-Aldrich) as a HUVEC culture medium and these cells were expanded on fibronectin-coated culture vessels.

### 4.3. (3-[4,5-Dimethylthiazol-2-yl]-2,5-Diphenyltetrazolium Bromides) MTT Assay

Cell proliferation and viability were determined via an MTT assay (Sigma-Aldrich). We suspended HUVECs and MSCs in culture vessels and cell lines were optimized as per the supplier’s suggestions. We then added arsenite with different dosages ranging from 0.3 to 60 μM, incubated the mixture for 48 h and then harvested cells for the MTT assay. Procedures were carried out according to the manufacturer’s instructions; we added 10 μL MTT labeling reagent and incubated the mixture for 4 h. The resulting solutions were transferred to 96-well plates and absorbance was recorded at 570 nm using a microplate spectrophotometer system (Spectra max190-Molecular Devices). The results are presented as percentages of control values.

### 4.4. Cytotoxicity and Apoptosis Assessment

Direct cytotoxicity tests were performed with a serial elution of arsenite (0, 5, 10, 15, 20 and 25 μm) added to the HUVEC and hMSC cell lines. We also designed a co-culture of HUVECs and hMSCs with Transwell plates and HUVECs with conditioned medium from hMSCs and arsenite. Each included group was inoculated into 6-well plates. At 24 h, the culture media was removed and different concentrations of arsenite were added with serum-free media and incubated for 24 and 48 h. Cell morphology was examined with a light microscope and the apoptosis rate was evaluated with Annexin V-FITC/PI staining (Abcam, Cambridge, MA, USA) and analyzed with flow cytometry. We collected 1 × 10^5^ cells by centrifugation, then suspended cells in 500 μL binding buffer. Cells were double stained with 5 μL Annexin F_FITC and 5 μL propidium iodide (PI), incubated at room temperature for 5 min and prepared for quantification with flow cytometry.

### 4.5. Cytokine Array Assay

A human cytokine antibody array kit (Abcam) was used to measure the cytokine production of hMSCs and HUVECs. Both cell lines were cultured alone and treated with 20 μm arsenite for 48 h. Supernatant was harvested from media and conditioned media from hMSCs. The array was performed according to the manufacturer’s instructions. Detection was carried out with biotin-conjugated antibodies and HRP-conjugated streptavidin, followed by an overnight incubation at 4 °C. Membranes were transferred to chromatography paper and chemiluminescence imaging was captured by a UVP BioSpectrum 600 System (UVP LLC, Upland, CA, USA).

### 4.6. Western Blotting

Supernatants from conditioned medium of hMSCs with 20 μM arsenite, incubated for 48 h, were collected for analysis. Twenty micrograms of protein were separated on a 12% polyacrylamide gel and transferred to a PVDF membrane. The membrane was blocked by 5% BSA (bovine serum albumin)/TBST (tris buffered saline with tween) for 1 h and then probed with primary antibodies of VEGF (Abcam) at 4 °C overnight. After TBST washing, the membrane was incubated with HRP (horseradish peroxidase)-conjugated secondary antibodies at room temperature for 2 h. After TBST washing, protein levels were detected using SuperSignal™ West Femto Maximum Sensitivity Substrate (Thermo Fisher Scientific). Chemiluminescence imaging was captured by a UVP BioSpectrum 600 System (UVP LLC, Upland, CA, USA).

### 4.7. Small Interfering (si) RNA Knockdown Test

The silencing of human VEGF was achieved by using PepMute™ siRNA transfection reagent (SignaGen Laboratories, Rockville, MD, USA). Transient transfection was performed in 6-well culture plates and carried out according to the protocol recommended by the manufacturer. Specifically, 5.0 nM siRNA was diluted in 100 μL transfection buffer and then mixed with 3 μL PepMute™ reagent. The mixed transfection complex then was added onto plates containing hMSCs and incubated for 72 h. HUVECs were also seeded on 6-well plates and then incubated for 24 h. A total of 20 μM arsenite, conditioned media from hMSCs, 20 μM As and conditioned media transfected with siRNA against VEGF were added separately to HUVECs, incubated for 48 h and then harvested for morphology and Annexin V apoptosis assays as described in Section 4.4.

### 4.8. Mitochondrial Test—Oxygen Consumption Rate (OCR) Test and Mitochondrial Mass Detection

Mitochondrial oxygen consumption rates (OCR) were measured with XF24 (Seahorse Bioscience Inc., Billerica, MA, USA). hMSCs and HUVECs were seeded on an XF24 culture plate; the experimental groups were treated with 20 μM arsenite. The optimal seeding density was 1 × 10^4^ cells/well and the cells were incubated for 48 h. We then continued the assay using the protocol described by the manufacturer. Oxygen consumption rates (OCR) were detected under basal conditions followed by sequential addition of oligomycin (line B in Figure 3A), carbonyl cyanide p-trifluoromethoxy-phenylhydrazone (FCCP) (line C in Figure 3A), as well as rotenone and antimycin A (line C in Figure 3A). Maximal respiratory rate was measured after cells were treated with FCCP (blue bar zone 29 in Figure 3A). Finally, data were calculated and generated automatically; data are presented as wave data for analysis.

Mitochondrial mass was measured with Acridine Orange 10-nonyl bromide (NAO, Sigma-Aldrich, USA). We prepared cell lines as described in the OCR test above. Cells at a sub-confluent stage were trypsinized and resuspended in 0.5 mL of phosphate-buffered saline (PBS, pH 7.4) containing 2.5 μM NAO. The harvested cells were incubated for 15 min at 25 °C in the dark, then immediately transferred to a tube for analysis on a flow cytometer (Model EPICS XL-MCL, Beckman-Coulter, Miami, FL, USA). The fluorescence intensity of 1 × 10^4^ cells was recorded on a flow cytometer with an excitation wavelength at 488 nm and emission wavelength at 535 nm.

### 4.9. In Vivo SD Rat Experiment

To assess the effect of As intoxication in vivo, we maintained SD rats purchased from the national animal center and maintained them with periodic 16 ppm As intraperitoneal injections once a week for 12 weeks and finally checked their serum As concentration. For the experimental group, mouse MSCs harvested from BALB/c mice were injected through the tail vein at the 12th week. Then, 1 × 10^6^ cells dispersed in 1 mL PBS solution were injected. Injections were repeated one week later and the total number of cells injected were 2 × 10^6^ cells over the course of two weeks. SD rats in the control and experimental groups were sacrificed one week after the last injection and we harvested specimens from the anterior compartment of hind limbs just above the ankle joint.

### 4.10. Apoptosis TUNEL Assay

Tissues containing the vascular bundle from the anterior lower limbs of SD rats were harvested for the detection of apoptosis. They were fixed in 10% formalin, embedded in paraffin and sectioned at 5 μM. After deparaffinization and rehydration, tissue sections were subsequently processed using a TUNEL apoptosis detection kit (R&D Systems, Minneapolis, MN, USA). Procedures were carried out according to the manufacturer’s instructions and then observed under a light microscope.

### 4.11. ELISA Test

Blood was drawn from SD rats, which were divided into wild-type, PBS injection only, As IP injection and mMSC injection groups. The VEGF concentrations in the blood were measured with a VEGF ELISA Kit (Abcam). The arrays were performed according to the manufacturer’s instructions. Blood samples were pipetted into 96-well plates, followed by the addition of biotinylated anti-rat VEGF antibody, HRP-conjugated streptavidin and TMB substrate solution. The absorbance levels of proteins were measured on a plate reader at 450 nm.

### 4.12. Glutathione GSH/GSSG Assay and SOD Assay

As previously mentioned, blood was drawn from SD rats and was then divided into wild-type, PBS injection only, As IP injection and mMSC injection groups. Whole blood was added to a tube and mixed with Bioxytech reduced glutathione and oxidized glutathione (GSH/GSSG) 412-kit (Oxis international Inc., Forster City, CA, USA) according to the manufacturer’s instruction. After centrifuging, the supernatant was extracted and processed for GSH/GSSG analysis. The absorbance levels of samples were measured on a plate reader at 412 nm. Activities of superoxide dismutase (SOD) were performed in blood samples by using colorimetric commercial kits, SOD ab65354 (Abcam, Cambridge, MA, USA). Plasma layers from blood were collected and then incubated in order to complete the enzyme reactions. The absorbance was measured at 450 nm.

### 4.13. Quantitative Real-Time PCR Analysis

RNA was prepared from tissue from the vascular bundle of the lower limbs of rats. Total RNA was isolated using TRIzol (Invitrogen, Carlsbad, CA, USA) and cleaned using an RNeasy Mini Kit (Qiagen, Courtaboeuf, France). We reverse transcribed the messenger RNA to complementary DNA using reagents (Genemark Technology, Taiwan) according to the manufacturer’s instructions. Quantitative real-time PCR analysis of total RNA from cultured cells was performed using the ABI Step One Plus Real-Time PCR System. cDNA was amplified using an ABI Step One Plus Real-Time PCR System at 95 °C for 60 s, 56 °C for 45 s and 72 °C for 60 s for 40 cycles, after initial denaturation at 95 °C for 5 min.

### 4.14. Data Analysis

Statistical analyses were performed by Student’s t test for two groups of data and by one-way analysis of variance (ANOVA) with post hoc tests for multiple comparisons. Data are expressed as the mean ± SEM from three independent experiments and *p* < 0.05 was considered statistically significant.

## 5. Conclusions

A preclinical model of cellular therapy using MSCs for the treatment of As-induced vascular diseases was established in this study. Our results indicate that adult stem cell MSCs therapy may provide a remarkable, feasible and effective treatment for overwhelming As intoxication. We also demonstrate that hMSCs are more resistant to As toxicity by preserving more functional mitochondria. Abundant VEGF secreted by MSCs under arsenite intoxication is of therapeutic efficacy and can support endothelial function and rescue endothelial cells from early apoptosis. The paracrine effect of MSCs under arsenite intoxication provides a therapeutic effect and ameliorates vascular abnormalities. Together, the translational application of MSCs into the treatment of As-induced vasculopathy deserves further investigation.

## Abbreviations

As	Arsenite
ROS	Reactive oxygen species
MSCs	Mesenchymal stem cells
hMSCs	Human mesenchymal stem cells
HUVECs	Human umbilical endothelial cells
LC50	Lethal concentration 50%
CM	Condition medium
Col-Cul	Co-cultured
ELISA	Enzyme-linked immunosorbent assay
VEGF	Vascular endothelial growth factor
Si	SiRNA for VEGF
OCR	Maximal oxygen consumption rate
SOD	Superoxidase dismutase
CD31	Platelet endothelial cell adhesion molecular
GSH	Reduced glutathione
GSSG	Oxidized glutathione
HE stain	Hematoxylin and eosin stain
SD rat	Sprague Dawley rat
TUNEL	Terminal deoxynucleotidyl transferase dUTP nick end labeling
